# Lead Exposure and Behavior among Young Children in Chennai, India

**DOI:** 10.1289/ehp.0900625

**Published:** 2009-06-26

**Authors:** Ananya Roy, David Bellinger, Howard Hu, Joel Schwartz, Adrienne S. Ettinger, Robert O. Wright, Maryse Bouchard, Kavitha Palaniappan, Kalpana Balakrishnan

**Affiliations:** 1 Department of Environmental Health Sciences, University of Michigan School of Public Health, Ann Arbor, Michigan, USA; 2 Department of Environmental Health, Harvard School of Public Health, Boston, Massachusetts, USA; 3 Department of Neurology, Children’s Hospital Boston, Boston, Massachusetts, USA; 4 Channing Laboratory, Brigham and Women’s Hospital, Boston, Massachusetts, USA; 5 Department of Medicine, Children’s Hospital Boston, Boston, Massachusetts, USA; 6 Department of Environmental Health Engineering, Sri Ramachandra Medical College and Research Institute, Chennai, Tamil Nadu, India

**Keywords:** ADHD, anxiety, blood lead, children, executive function, India, sociability

## Abstract

**Background:**

Lead exposure has long been associated with deficits in IQ among children. However, few studies have assessed the impact of lead on specific domains of behavior and cognition.

**Objective:**

We evaluated the associations between lead and different domains of neurobehavior and their relative sensitivity to lead.

**Methods:**

We determined blood lead levels using a LeadCare instrument in 756 children 3–7 years of age attending pre- and elementary schools in Chennai, India. Anxiety, social problems, inattention, hyperactivity, and attention deficit hyperactivity disorder (ADHD), as well as executive function were assessed in children by their schoolteachers using Conners’ Teacher Rating Scales-39, Conners’ ADHD/Diagnostic and Statistical Manual for Mental Disorders, 4th Edition Scales (CADS), and the Behavior Rating Inventory of Executive Function questionnaires, with higher scores denoting worse behavior. Analyses were carried out using multivariate generalized estimating equations with comparisons of outcome *Z*-scores to assess the relative strengths of the associations between log-blood lead and the different domains of behavior.

**Results:**

Mean blood lead level was 11.4 ± 5.3 μg/dL. Blood lead was associated with higher anxiety (β = 0.27, *p* = 0.01), social problems (β = 0.20, *p* = 0.02), and higher scores in the ADHD index (β = 0.17; *p* = 0.05). The effect estimate was highest for global executive function (β = 0.42; *p*< 0.001).

**Conclusions:**

Higher blood lead levels in this population of young children is associated with increased risk of neurobehavioral deficits and ADHD, with executive function and attention being particularly vulnerable domains to the effects of lead.

The removal of lead from sources in the environment, such as gasoline and residential paint, have resulted in declines in blood lead levels in many countries [Centers for Disease Control and Prevention (CDC) 1997, 2005a]. In India, leaded gasoline was phased out in early 2001. Nichani et al. (2006) reported that 37% of children in Mumbai in 2002 had blood lead levels > 10 μg/dL, the CDC level of concern for blood lead (CDC 2005b), down from 60% in 1997. Other studies describe continued elevations in blood lead levels among children in other parts of the country (Ahamed et al. 2006; Bellinger et al. 2005).

Many studies have shown that lead exposure is associated with significant deficits in intelligence quotient (IQ) of children (Lanphear et al. 2005). However, IQ is a global construct of cognition, and fewer studies have assessed the impact of lead on specific domains of behavior and cognition, such as executive function (Canfield et al. 2003, 2004; Froehlich et al. 2007), attention (Bellinger et al. 1994; Stiles and Bellinger 1993), and internalizing behaviors. Recent evidence suggests that increased lead exposure is associated with attention deficit hyperactivity disorder (ADHD) (Braun et al. 2006; Nigg 2008) and conduct disorder (Needleman et al. 2002; Nevin 2000). Currently, relatively little is known about the shape of the dose–response relationships or the relative sensitivity of behavioral domains to lead toxicity. Only one study has reported on the relationship between lead exposure and behavior, as well as other cognitive outcomes among children in India (Bellinger et al. 2005).

The objective of this study was to explore the associations between lead and specific neurobehavioral outcomes such as ADHD-like behaviors, executive function, and internalizing problems (anxiety and sociability) in a cohort of children 3–7 years of age in Chennai, India. In addition, we investigated the shape of the dose–response relationships and the relative sensitivity of the different behavioral outcomes to lead exposure.

## Methods

### Study population

This cross-sectional study of 814 children was carried out during 2005–2006 in Chennai, India. Four traffic and industry zones were selected on the basis of industrial zoning information provided by the Tamil Nadu State Pollution Control Board and the Chennai traffic police department. Children 3–7 years of age were recruited from 12 schools (three schools within each zone). Informed consent was obtained from each child’s primary caregiver. Venous blood was collected from each child, and questionnaires were administered in Tamil (the regional language) to the teachers and primary caregivers (usually the mothers) of the children. A semi-quantitative food frequency questionnaire was used to assess nutritional status. Information was collected on a family’s average monthly income, educational attainment and occupation of the parents, and type of housing. Data on family size, maternal age at birth of child, and child’s age, birth weight, and birth rank were also collected.

This investigation was conducted as part of a collaborative study between the Harvard School of Public Health (HSPH), Boston, Massachusetts (USA), and Sri Ramachandra Medical College and Research Institute (SRMC), Chennai, India, on lead genetics and neurotoxicity. The study was approved by institutional review boards at HSPH, SRMC, the University of Michigan, and the Indian Council for Medical Research, New Delhi, India, and complied with U.S. federal and Indian regulations and laws regarding research on human subjects.

### Blood lead measurement

Blood was collected from the cubital vein of each child into lead-free tubes (royal blue top tubes #369736; Becton-Dickinson, Franklin Lakes, NJ, USA) by a trained phlebotomist during a school day. The blood was transferred to the laboratory at SRMC, and blood lead was measured using Anodic Stripping Voltammetry by a LeadCare Analyzer (ESA Laboratories, Chelmsford, MA, USA), which is a well-validated field instrument with sensitivity of 1 μg/dL blood lead (Shannon and Rifai 1997). Duplicates and controls were run with every 20 samples, every new batch, and kit lot. Fifty-eight children were sick or absent from school, rendering us unable to collect a blood sample; the final sample comprised 756 children with all measurements (92% of enrolled population).

### Neurobehavior assessment

Behavioral outcomes were assessed through administration of the following questionnaires, translated into Tamil, to the class teacher of each child: *a*) the Conners’ ADHD/Diagnostic and Statistical Manual for Mental Disorders, 4th edition (DSM-IV) Scales (CADS-T) (Conners et al. 1998); *b*) an older version of the Conners’ Teachers Rating Scales (CTRS-39) (Conners 1990) that continues to be widely used in India; and *c*) the Behavior Rating Inventory of Executive Function (BRIEF) (Gioia et al. 2000). For all questionnaires, higher scores indicate worse behaviors.

The CADS-T is a self-administered questionnaire for teachers comprising 27 questions that assess behaviors associated with ADHD. It yields three scores: ADHD index score, DSM-IV inattentive-impulsive subscale, and DSM-IV hyperactive subscale. The ADHD index was empirically derived from the 12 items best differentiating children with ADHD from nonclinical children in several large data sets. The items that comprise the DSM-IV inattentive-impulsive and hyperactive scales correspond to the DSM-IV criteria for ADHD diagnosis.

The CTRS-39 consists of 39 questions used to assess internalizing and externalizing dimensions of behavior with five subscales: aggressivity, inattentiveness, anxiety, hyperactivity, and sociability. However, because this is an older version of the CTRS-39, we chose to use only the subscales that did not overlap with those of the CADS-T, namely, anxiety and sociability.

The BRIEF questionnaire for teachers was used to assess the children’s executive function. The BRIEF is useful in evaluating children 5–18 years of age with a wide range of developmental and acquired neurologic conditions such as learning disabilities, ADHD, and traumatic brain injury. This 86-item questionnaire takes 10–15 min to complete. Following the scoring procedure from the test manual (Gioia et al. 2000), the questionnaire responses were used to compute a global executive composite score and eight nonoverlapping clinical scales within the two broader indexes: *a*) behavioral regulation index (three scales: inhibit, shift, and emotional control), and *b*) metacognition index (five scales: initiating, working memory, plan/organize, organization of materials, and monitor). These scales were derived theoretically and empirically, and good convergent validity was observed with other measures of inattention, impulsivity, and learning skills (Gioia et al. 2000).

### Data analysis

We examined descriptive statistics for all variables. Blood lead was natural log-transformed to maintain normality of the residuals in the model. Outliers (*n* = 9) were identified using extreme studentized deviate (Rosner 1983). We performed all analyses with and without the outliers. In general, the results did not change appreciably after removal of the outliers from the final multivariate models.

We used analysis of variance for bivariate analysis to explore associations and potential confounders. We performed preliminary analyses and model building using multivariate linear regression. Covariates were selected in the regression procedure if they changed the *R*^2^ and/or main effect estimate by > 10%. On the basis of biological premise and from previous literature, parental education and hemoglobin were included in the models.

Because the study had a nested hierarchical sampling structure, linear models would have narrower standard errors due to correlation between observations at every level of sampling and within the neurobehavioral outcomes of students assessed by common teachers. We examined the intraclass correlation of behavioral outcomes at each level (zone, school, class) using random effects mixed models. Because most outcomes were not normally distributed and covariance within the levels did not fit a specified structure, we chose to account for the clustering of observations in the marginal models with generalized estimating equations (GEE) which use quasi-likelihood methods for estimation (Qaqish and Liang 1992).

Scores on the different subscales of the CADS-T and CTRS were analyzed separately. For executive function, the BRIEF global executive composite score was analyzed first, and because a significant association was found, further analyses were then carried out on the subscale scores.

Because there are no normative data on these questionnaires for Indian children, we standardized raw scores internally by computing *Z*-scores with respect to sex and age (similar to the process used to create *T*-scores in psychometric tests). Analyses were carried out on the resulting *Z*-scores and compared with the analyses using raw scores to determine the consistency of the results.

To ascertain whether the associations with blood lead differed across neurobehavioral domains (anxiety, sociability, ADHD index, and global executive composite score), we treated the *Z*-scores as repeated measures of neurobehavior and added an indicator (dummy) variable for each neurobehavioral domain. The association between blood lead and a particular neurobehavioral domain was considered significant if the interaction term for lead and the dummy-coded domain variable had a *p* < 0.05. Similar analyses were carried out to investigate differences between BRIEF subscales of executive functions and between the CADS-T ADHD index score, DSM-IV inattentive and hyperactivity subscales.

We explored dose–response relationships using generalized additive models within mixed models, accounting for clustering, using R statistical software (Free Software Foundation Inc., Boston, MA, USA). When nonlinearity was present, the penalized spline model was compared with the linear model using Akaike’s information criterion and generalized cross-validation.

## Results

Mean (± SD) blood lead was 11.4 ± 5.3 μg/ dL (range, 2.6–40.5) and 54.5% of the children had blood lead levels > 10 μg/dL. Blood lead was associated with markers of socioeconomic status, with higher blood lead observed among those who had lower average family monthly income [compared with those who made > 13,000 rupees/month (US$518)] and greater number of children in the family. Significantly lower blood lead levels were noted among children whose parents had finished college (Table 1).

Bivariate analyses indicated a trend of higher (i.e., worse) behavioral scores with increasing blood lead concentrations (Table 2).

The variance in neurobehavioral outcomes, assessed using a random effects model, was significantly associated within schools and classrooms, indicating correlation. Multivariate GEE analyses, accounting for clustering at the class and school level and controlling for child’s age (months), sex, hemoglobin level, family average monthly income, maternal and paternal education, and number of children in the family, showed that higher blood lead level was associated with higher behavioral raw and *Z*-scores (indicating worse behavior). The use of *Z*-scores versus raw scores did not change the overall direction or significance of the associations (Table 3). Nevertheless, the use of *Z*-scores allowed for standardization by age and sex, and for direct comparison of beta coefficients for different test scores.

On the CTRS-39, a one-unit increase in log blood lead was associated with 0.27 higher anxiety *Z*-score (*p* = 0.017) and 0.20 higher sociability *Z*-score (*p* = 0.027). On the CADS-T, blood lead was associated with higher ADHD index *Z*-scores and DSM-IV inattentive, (β = 0.17, *p* = 0.05 and β = 0.24, *p* = 0.01, respectively) but not hyperactive. On the BRIEF, the global executive composite score was strongly associated with higher blood lead level (*p* < 0.001).

**Figure 1:**
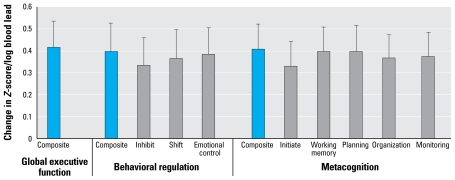
Differing sensitivity (change in *Z*-score/unit log blood lead) of executive function subscales (BRIEF) to lead exposure. Error bars indicate SE.

In comparing the response of the behaviors, we found that change in executive function with blood lead was significantly higher than that of ADHD index, anxiety, or sociability (*p* < 0.001), with a change of 0.42 *Z*-scores per increase in one unit of log blood lead. Within the behavioral domains of ADHD, a one-unit change in log blood lead was associated with an increase of 0.24 *Z*-score of attention compared with 0.13 points of *Z*-score of hyperactivity and was significantly different (*p* < 0.05). There were no significant differences between the effects of lead within the different domains of executive function (Figure 1).

**Figure 2:**
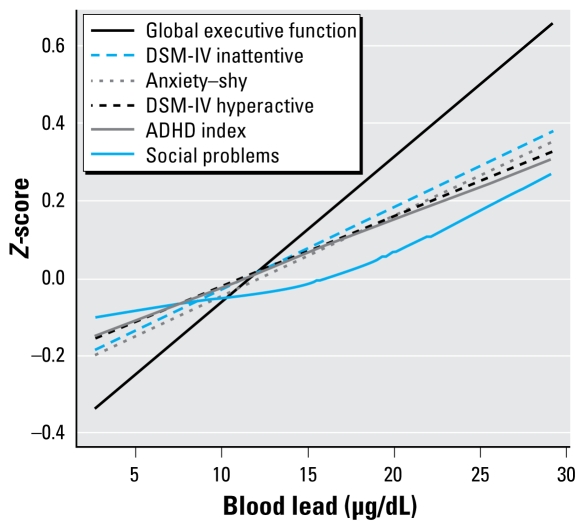
Dose–response relationship between blood lead and behavior. Smooth functional term relating behavior *Z*-scores to blood lead in an adjusted generalized additive mixed model, adjusting for age, sex, hemoglobin, average monthly income, maternal and paternal education, and number of other children and accounting for clustering at school and classroom level. Social problems were not significantly nonlinear (effective degrees of freedom = 1.56).

The dose–response relationships for lead and all behavioral outcomes, assessed by cubic penalized splines, were linear. No inflection points, indicating particular threshold levels, were noted in any of the associations (Figure 2). There was no significant difference in the effect of lead above and below 10 μg/dL or effect modification by sex.

## Discussion

Lead levels in this population were higher than currently seen in many countries (CDC 1997, 2005a), and higher blood lead levels among young children were associated with deficits across a wide range of behavioral outcomes. Our study is among the first to note that lead exposure is associated with increased specific childhood internalizing behaviors such as anxiety and social problems. We also observed that children with higher blood lead levels presented with more ADHD-type behaviors, especially the inattention component, which builds upon the nascent literature in this area (Braun et al. 2006; Wang et al. 2008). Of particular interest is the finding of a larger effect of lead on executive function and attention compared with the other behaviors assessed. The dose response in this population failed to identify a threshold level for the association between lead and the behaviors. Specifically, we found no evidence that the associations departed from linearity.

The association of blood lead level with anxiety and poor sociability among children is important. The children with higher anxiety and sociability scores in the CTRS-39 were those who had low self-esteem and little self-confidence; were withdrawn, shy, emotionally distant, and detached from their peers; and also tended to worry and to be emotional and sensitive to criticism as well as being particularly anxious in new or unfamiliar situations. Such behaviors may lead to functional isolation, which is hypothesized to decrease exploration of the environment and may make children less likely to seek and/or receive attention and nurturing stimulation, such as developmentally facilitating care from their caregivers (teachers/parents) through responsiveness and verbal stimulation (Lozoff 1998; Walter 1989).

There are very few studies that describe a statistically significant effect of lead on anxiety in children. A few studies have reported increased internalizing behaviors among children exposed to lead, but except for the study by Burns et al. (1999), most did not find significant associations with different types of internalizing behaviors (Sciarillo et al. 1992; Wasserman et al. 2001). A study by Bellinger et al. (1994) reported increased internalizing behavior and noted higher anxiety among children. Evidence from animal research indicates that early lead exposure causes permanent changes in the hypo-thalamic–pituitary axis, glucocorticoid dysregulation, and altered dopaminergic and GABA-ergic (γ-aminobutyric acid–containing) systems, which are associated with increased anxiety and decreased socializing behavior (Nieto-Fernandez et al. 2006). These pathways are also potentiated by stress (Cory-Slechta et al. 2008; White et al. 2007), which may be more prevalent among lower socioeconomic populations.

ADHD refers to a constellation of symptoms including difficulty in delaying gratification, overactivity or motor restlessness, distractibility, impulsivity, aggression, and short attention span (Spreen et al. 1995). In the current study, children with higher lead exposures had higher prevalence of ADHD-like behaviors (marginally significant). This is consistent with a recent analysis of the National Health and Nutrition Examination Survey by Braun et al. (2006), which showed that the odds of diagnosed ADHD were 4.1 times higher among children within the highest quintile of concurrent blood lead level than among those in the lowest quintile of lead exposure. These results were corroborated by results from other studies among children (Millichap 2008; Tuthill 1996; Wang et al. 2008). However, other studies have not differentiated between inattention and hyperactive-type ADHD. In this study we found that blood lead was strongly associated with behaviors reflecting predominantly inattention rather than hyperactivity. In fact, lead was not significantly associated with the latter in this population. Previous studies have shown the impact of lead on attention, and that among older children and adolescents, processes regulating attention may be the underlying cause (Bellinger et al. 1994; Burns et al. 1999; Davis et al. 2004). Animal studies also indicate that lead affects processes regulating attention such as impulsivity (Brockel and Cory-Slechta 1998).

During the last decade there has been increasing interest in executive function in childhood. Executive function is an umbrella term that incorporates a collection of interrelated processes responsible for purposeful, goal-directed behavior (Anderson 2002). This construct includes cognitive control processes such as planning, regulation of attention (resistance to distraction), decision making, working memory, problem solving, and behavioral control (impulse control, initiation and monitoring of action, including self-monitoring, and shifting from task to task) (Arnsten and Li 2005; Mitchell and Phillips 2007).

The association between lead exposure and executive function—such as metacognitive functioning involving working memory, planning, and organization—confirms the finding of poorer functioning in task clusters among schoolchildren with higher prenatal blood lead, which suggests these deficits (Leviton et al. 1993). Studies using the digit span test as a measure of working memory have suggested a detrimental effect, as seen in some occupational studies (Hanninen et al. 1998; Stewart et al. 2002). Few studies have focused specifically on executive function in young children.

Canfield et al. (2004), using the Cambridge Neuropsychological Testing Automated Battery in a population of 174 children exposed to low levels of lead, suggested that lead exposure affects working memory, attentional flexibility, planning, and problem solving. However, this association was not supported by another study in 170 preschool children (Canfield et al. 2003) in which the authors reported a significant association with deficits in performance, attention, and the inhibition of automatic responses, but failed to find consistent effects on tasks requiring attention switching and the combination of inhibition and switching. This result may be attributable to the difficulty of the test administered and the relatively low blood lead levels of the children tested.

Our study demonstrates that, across the range of exposure in this population of schoolchildren in India and using a measure that focuses on observed behavioral regulation in daily life, higher blood lead levels are associated with deficits across multiple domains of executive function (including inhibition of automatic responses and attention shifting). This finding is consistent with studies that report significant associations with perseverative errors (indicating an inability to shift attention) in the Wisconsin Card Sorting Test and the California Verbal Learning Test (Stiles and Bellinger 1993; Surkan et al. 2007).

The results of this study are consistent with animal data showing lead exposure–induced changes in myelination, oxidative stress, cellular signaling, and multiple neurotransmitter pathways related with behavior and executive function. Studies by Cory-Slechta (2003) suggest that lead affects the dopaminergic pathway and that these changes are connected to changes in perseveration and impulsivity errors in animals. There is also considerable evidence that the glutamatergic system, specifically the *N*-methyl -aspartate (NMDA) receptor complex, which is associated with learning and memory, is affected by lead exposure in animals (Finkelstein 1998).

This study has several limitations. The cross-sectional design precludes consideration of the temporal relationships between exposure and outcome. It is possible that behavioral changes precede lead exposure and could even induce lead exposure through behavioral pathways such as increased hand-to-mouth behavior. The lack of validation of the BRIEF, CADS, and CTRS within the Indian population is another potential limitation. We ensured internal validity in this study by using *Z*-scores, standardized within the population. Nevertheless, caution must be exercised in the generalizability of the reported absolute magnitudes of effect estimates, as the effect of lead within the Indian population may be modified by sociocultural, genetic, and nutritional factors particular to the Indian subcontinent. Residual confounding in the relationship between lead and neurobehavior could exist, because it was not feasible to assess home environment and parental behavior or IQ. However, we did control for family size, socioeconomic status, and maternal education, all of which are correlated with a nurturing environment for a child (Seifer 2001). The association of lead with anxiety and sociability may reflect residual confounding due to co-occurrence of high-stress environments and high lead exposures associated with low socioeconomic status. However, in all analyses, we accounted for socioeconomic status using three measures (income, family size, and parental education). Of course, there is always a chance that some confounding remains, and it is possible that the effects of lead and stress may be synergistic in affecting anxiety and social behavior in young children (Cory-Slechta et al. 2008; White et al. 2007).

Despite the limitations, this is the first large, population-based study of lead and neurobehavior to be carried out in India and the first study to assess differential sensitivity of the effects of lead on behavior. The use of the BRIEF questionnaire allows assessment of executive function in 3- to 7-year-old children as observed by teachers in tasks required in a regular school day compared with other studies that use computer-based tasks, which have often proven too difficult for young children.

## Conclusion

Overall, this study suggests that lead exposure affects behavior across multiple domains, including anxiety and social behavior. The results also suggest that executive functions and attention are especially vulnerable to insult by lead among young children. We identified no threshold for these effects, and all dose–response relationships were linear. This is an important finding for policy decision making, as it suggests that there might be no safe level of lead exposure.

In terms of societal disease burden, the behavioral and psychiatric morbidities associated with lead might be even more important than cognitive morbidities (Bellinger 2001). A conservative estimate of cost of illness of ADHD among children and adolescents in the United States in 2005 amounted to $42.5 billion/year, assuming a 5% prevalence in the country (Pelham et al. 2007).

This may have policy implications for a country such as India that is rapidly undergoing industrialization, where elevated childhood lead exposures are prevalent, and where 31% of the population is < 15 years of age (National Health and Family Survey 2001) and vulnerable to the long-term effects of lead exposure. These findings likely have implications not only for the Indian population, but for other populations in developing countries where similar lead exposure scenarios exist.

## Figures and Tables

**Table 1:** Blood lead levels in relation to individual characteristics among young children in Chennai, India.

	Blood lead (μg/dL)	
Characteristic	No. (%)	Mean ± SD
Age (years)		
3	127 (16.8)	10.80 ± 5.36
4	246 (32.5)	11.54 ± 5.49
5	248 (32.8)	11.57 ± 5.17
> 6	135 (17.9)	11.81 ± 5.32
Sex		
Male	404 (53.4)	11.57 ± 5.35
Female	352 (46.6)	11.37 ± 5.32
No. of other children in family		
0	168 (22.2)	10.56 ± 4.68
1	483 (63.9)	11.61 ± 5.41*
2	82 (10.8)	12.82 ± 5.93**
> 3	23 (3.0)	10.48 ± 4.91
Household average monthly income (rupees)		
< 4,000	445 (58.9)	12.14 ± 5.49**
4,000–13,000	272 (36.0)	10.88 ± 5.06**
> 13,000	39 (5.2)	8.06 ± 3.18
Mother’s educational attainment		
Illiterate/primary school	139 (18.4)	11.61 ± 5.28
Middle school	254 (33.6)	11.99 ± 5.36
High school certificate	256 (33.9)	11.39 ± 5.25
College	107 (14.2)	10.28 ± 5.42*
Father’s educational attainment		
Illiterate/ primary school	95 (12.6)	11.98 ± 5.36
Middle school	213 (28.2)	12.28 ± 5.14
High school certificate	298 (39.4)	11.34 ± 5.5
College	150 (19.8)	10.29 ± 5.05**
Iron supplementation		
No	671 (90.1)	11.58 ± 5.36
Yes	74 (9.90)	10.76 ± 5.31
Calcium supplementation		
No	665 (89.4)	11.52 ± 5.26
Yes	79 (10.6)	10.66 ± 5.11

* *p* < 0.05.

** *p* < 0.01.

**Table 2:** Behavioral raw scores by quartiles of blood lead (mean ± SD).

	Quartiles of blood lead (μg/dL)			
	Q1	Q2	Q3	Q4
Behavioral scales	6.13 ± 1.16	9.06 ± 0.78	12.10 ± 1.10	18.71 ± 4.80
CTRS				
Anxiety–shy	1.65 ± 0.57	1.74 ± 0.64	1.76 ± 0.60	1.85 ± 0.65
Social problems	1.40 ± 0.65	1.49 ± 0.68	1.44 ± 0.64	1.51 ± 0.68
CADS-T				
ADHD index	11.57 ± 8.2	11.59 ± 8.56	11.49 ± 9.08	13.71 ± 8.67
DSM-IV inattentive	7.88 ± 6.14	8.4 ± 6.96	8.39 ± 6.84	10.29 ± 7.26
DSM-IV hyperactivity	8.96 ± 6.4	9.35 ± 6.60	8.89 ± 6.56	10.3 ± 6.60
BRIEF				
Global executive	112.55 ± 29.14	118.15 ± 31.63	120.15 ± 28.77	129.83 ± 33.03
Behavioral regulation	44.69 ± 11.65	46.73 ± 12.72	48.28 ± 11.67	51.05 ± 12.66
Metacognition	67.86 ± 18.18	71.43 ± 19.60	71.87 ± 18.21	78.77 ± 21.19

**Table 3:** Comparison of multivariate^a^ GEE analysis of log blood lead and behavior (raw scores and *Z*-scores).

	Raw scores		*Z*-scores^b^	
Behavioral scales	β (95% CI)	*p*-Value	β (95% CI)	*p*-Value
CTRS-39				
Anxiety–shy	0.17 (0.03 to 0.31)	0.020	0.27 (0.05 to 0.51)	0.017
Social problems	0.14 (0.02 to 0.26)	0.025	0.20 (0.02 to 0.38)	0.027
CADS-T				
ADHD index	1.45 (–0.10 to 3.01)	0.066	0.17 (–0.00 to 0.36)	0.053
DSM-IV inattentive	1.67 (0.38 to 2.98)	0.012	0.24 (0.05 to 0.43)	0.012^c^
DSM-IV hyperactive	0.87 (–0.26 to 1.99)	0.129	0.13 (–0.04 to 0.30)	0.126
BRIEF				
Global executive function	12.96 (5.46 to 20.45)	0.001	0.42 (0.18 to 0.65)	0.001^d^

CI, confidence interval.

a Adjusting for age, sex, hemoglobin, average monthly income, maternal and paternal education, number of other children and accounting for clustering at school and classroom level.

b Created by standardizing raw scores by age (3–5/6–7 years) and sex-stratified mean and SD.

c The effect of lead on attention is significantly larger that the effect on hyperactivity (*p* < 0.05).

d Global executive function (BRIEF) is significantly (*p* < 0.001) more sensitive to the effect of lead compared with ADHD, anxiety, and social problems.
